# HRG-β1-driven ErbB3 signaling induces epithelial–mesenchymal transition in breast cancer cells

**DOI:** 10.1186/1471-2407-13-383

**Published:** 2013-08-12

**Authors:** Jinkyoung Kim, Hoiseon Jeong, Youngseok Lee, Chungyeul Kim, Hankyeom Kim, Aeree Kim

**Affiliations:** 1 Department of Pathology, Korea University Guro Hospital, #97 Gurodong-gil, Guro-gu, Seoul 152-703, Korea; 2 Department of Pathology, Cheil General Hospital and Women’s Healthcare Center, 1-19 Mukjeong-dong, Jung-gu, Seoul 100-380, Korea

**Keywords:** Heregulin, Transforming growth factor-β (TGF-β), Smad2, EMT, Breast cancer, ErbB3, Small interfering RNA (siRNA)

## Abstract

**Background:**

Heregulin (HRG; also known as neuregulin) is a ligand for ErbB3. One of its isotypes, HRG-β1, binds to ErbB3 and forms heterodimers with other ErbB family members, thereby enhancing the proliferation and tumorigenesis of breast cancer cells. HRG stimulation may contribute to the progression of epithelial–mesenchymal transition (EMT) and tumor metastasis in breast cancer. Majority of studies regarding EMT has been concentrated on TGF-β signaling. Therefore, we investigated whether the HRG-β1 and ErbB3 activate Smad2 signaling during process of EMT in breast cancer cells.

**Methods:**

The SK-BR-3 and MCF7 breast cancer cell lines were used. The expressions of phospho-Smad2 and EMT markers were observed by western blotting and immunofluorescence assays after treatment with HRG-β1. The cell motility and invasiveness were determined by wound healing and matrigel invasion assays. Smad2 and ErbB3 small interfering RNA (siRNA) transfections were performed to assess the involvement of ErbB3 and Smad2 in HRG-β1-induced EMT.

**Results:**

HRG-β1 induced EMT through activation of Smad2. The expression of E-cadherin was decreased after HRG-β1 treatment, while the expressions of Snail, vimentin, and fibronectin were increased. The HRG-β1-induced expressions of Snail, vimentin, and fibronectin, and nuclear colocalization of phospho-Smad2 and Snail were inhibited by pretreatment with a PI3k inhibitor, LY294002, or two phospho-Smad2 inhibitors, PD169316 or SB203580 and cancer cell migration by HRG-β1 was inhibited. Knockdown of Smad2 by siRNA transfection suppressed the expressions of Snail and fibronectin in response to HRG-β1 stimulation and knockdown of ErbB3 suppressed the expressions of phospho-Smad2, Snail, and fibronectin induced by HRG-β1, whereas E-cadherin was increased compared with control siRNA-transfected cells. Knockdown of ErbB3 and Smad2 also decreased SK-BR-3 and MCF7 cell invasion.

**Conclusions:**

Our data suggest that HRG-β1 and ErbB3 induce EMT, cancer cell migration and invasion through the PI3k/Akt-phospho-Smad2-Snail signaling pathway in SK-BR-3 and MCF7 breast cancer cells.

## Background

Epithelial–mesenchymal transition (EMT) is a highly conserved and fundamental process that governs morphogenesis in multicellular organisms. EMT is involved in both embryonic development and progression of carcinoma toward dedifferentiated and more malignant states [1]. It is defined by loss of the epithelial phenotype and acquisition of mesenchymal characteristics, such as migratory capacity, loss of polarity, and cell-to-cell contacts [2]. EMT can contribute to tumor invasion, metastasis, and resistance to specific chemotherapy or hormone therapy.

EMT can be triggered by different signaling molecules, such as epidermal growth factor (EGF), fibroblast growth factor, hepatocyte growth factor, transforming growth factor (TGF)-β, bone morphogenetic proteins, WNTs, and Notch [3]. Among them, TGF-β is a major inducer of EMT [4,5]. Binding of TGF-β to its receptor leads to activation of the transcription factors Smad2/3, which form complexes with Smad4 and then translocate into the nucleus, where they control the transcription of target genes [6] in collaboration with specific transcription factors and cofactors such as Snail, Slug, and Zeb1/2 [7,8]. In particular, the role of the Snail family of zinc finger proteins in EMT and cancer has been highlighted in several publications [9,10].

Heregulin (HRG; also known as neuregulin) is a member of the EGF-like growth and differentiation factors, and binds with high affinity to the receptors ErbB3 and ErbB4 [11]. ErbB3, a member of the human epidermal growth factor receptor (EGFR) family of transmembrane receptors, undergoes heterodimerization with other ErbB family members and leads to cell differentiation, migration, proliferation, and survival [12]. Although four genes have been identified (HRG1–4), most research interests have focused on the HRG1 gene [13].

HRG-1 has been implicated in normal heart and nervous system development [14] as well as in the pathophysiological processes of psychiatric diseases, cardiac diseases, and various types of cancer [15,16]. HRG-1 is expressed in 30% of human breast cancer patients [17] and is correlated with poor histological grades [18]. Cheng et al. [19] demonstrated that HRG-β1 induced EMT through upregulation of Snail via the PI3k/Akt pathway in the SK-BR-3 cell line. However, the mechanism of HRG-β1 and ErbB3 for the regulation of EMT in breast cancer cells has not been documented in detail. In this study, we investigated whether HRG-β1/ErbB3 induces the process of EMT with involvement of Smad2 activation in the ErbB2-overexpressing SK-BR-3 cell line and luminal A breast cancer cell line MCF7.

## Methods

### Cell lines and culture

The human breast cancer cell lines SK-BR-3 and MCF7 were purchased from the American Type Culture Collection (ATCC, Manassas, VA). The cells were maintained in RPMI-1640 medium (GIBCO, Grand Island, NY) supplemented with 10% fetal bovine serum, 100 U/ml penicillin, and 100 mg/ml streptomycin (GIBCO). Both cell lines were cultured in a 37°C humidified atmosphere containing 95% air and 5% CO_2_.

### Reagents and antibodies

Recombinant human HRG-β1 (purity: >97%) was purchased from R&D Systems (Minneapolis, MN). It was divided into small aliquots in phosphate-buffered saline (PBS) and stored at –70°C. The PI3k inhibitor, LY294002 and phospho-Smad2 pharmacological inhibitors, PD169316 and SB203580 were purchased from Calbiochem (San Diego, CA). The inhibitors were dissolved in dimethyl sulfoxide (DMSO). An anti-ErbB3 antibody was purchased from Santa Cruz Biotechnology Inc. (CA, USA). Anti-phospho-Smad2 (Ser465/467) and anti-Smad2 antibodies were purchased from Cell Signaling Technology Inc. (Beverly, MO). An anti-Snail antibody was obtained from Abcam Ltd. (Cambridge, UK). Anti-E-cadherin and anti-vimentin antibodies were from BD Pharmingen (San Diego, CA). An anti-fibronectin antibody was obtained from Millipore (Billerica, MA). A monoclonal anti-β-actin antibody was obtained from Sigma (St Louis, MO).

### Western blotting

Cells were harvested and lysed with RIPA buffer (20 mM Tris–HCl pH 7.5, 2 mM EDTA, 150 mM NaCl, 1 mM sodium vanadate, 10 mM NaF, 2.5 mM sodium pyrophosphate, 1% sodium deoxycholate, 0.1% SDS, 1% NP-40) supplemented with a protease inhibitor (1 mM phenylmethylsulfonyl fluoride) and a protease inhibitor cocktail (Roche, Mannheim, Germany). The cell lysates was cleared by centrifugation at 14,000 rpm for 20 min at 4°C, and the supernatants were used as total cellular protein extracts. The protein concentrations were determined using a BCA protein assay kit (Pierce, Rockford, IL). The protein lysates were resolved by sodium dodecyl sulfate-polyacrylamide gel electrophoresis and then transferred to polyvinylidene fluoride membranes (Bio-Rad Laboratories, Hercules, CA). The blocked membranes with 5% skim milk were incubated with the indicated primary antibodies, followed by incubation with horseradish peroxidase-labeled secondary antibodies. Antibody-bound proteins were detected using the Enhanced Chemiluminescence (ECL) reagent (Amersham Biosciences, Piscataway, NJ) according to the manufacturer's instructions. The levels of protein expression were quantified using ImageJ software (NIH, Bethesda, MD) and then normalized by the corresponding expression level in control cells for each group.

### Immunofluorescence

Nuclear translocation of phospho-Smad2 and Snail was examined by immunofluorescence staining. Approximately 2 × 10^4^ cells/well were seeded onto 2-well Lab-Tek II chamber slides (NUNC, Rochester, NY). After serum starvation, the cells were incubated with HRG-β1 and specific inhibitors. The cells were then washed three times with PBS and fixed with 4% paraformaldehyde for 10 min. Following three washes with PBS, the cells were permeabilized with 0.1% Triton X-100 for 20 min. After washing with PBS, the cells were blocked with 3% bovine serum albumin for 1 h at room temperature and then incubated with rabbit polyclonal anti-Snail (1:500) and anti-phospho-Smad2 (1:100) primary antibodies overnight at 4°C. After three washes with PBS, the cells were incubated with Alexa Fluor 488-conjugated anti-rabbit IgG and Alexa Fluor 594-conjugated anti-goat IgG secondary antibodies (Invitrogen, Grand Island, NY). The cells were then washed, mounted with mounting medium containing DAPI (VECTOR Laboratories, Burlingame, CA), and observed using an LSM700 confocal laser scanning microscope (Carl Zeiss, Thornwood, NY). The expressions of E-cadherin and vimentin were evaluated with specific antibodies as described above and incubated with a DyLight 488-conjugated anti-mouse IgG secondary antibody (VECTOR Laboratories).

### Wound healing assay

For scratch wound healing assays, cells were seeded into 12-well plates and grown to confluence. After serum starvation, the confluent monolayers were scratched with a plastic tip, washed with PBS to remove the detached cells, and incubated with HRG-β1 and the indicated inhibitors for 24 h. The cell migration into the wounded area was monitored at the indicated time points using a light microscope (Olympus BX51 Tokyo, Japan). Quantification of the closure of the monolayers was determined using an NIH image analysis program and the results were presented as the relative percentages of wound closure compared with control monolayers. The assays were repeated three times independently.

### Matrigel invasion assay

For invasion assay, serum free medium (500 μl) treated with or without HRG-β1 was added to the lower chambers of a 24 transwell plate (8.0 μm pore size, Corning, NY) and untransfected or transfected with control (Ctrl), Smad2 and ErbB3 siRNA cells (2 × 10^5^ cells in 200 μl medium) were seeded in upper chamber which was coated with Matrigel (BD Biosciences). After 48 h of incubation, non-migrating cells were removed with a cotton swab and cells on the bottom surface of the membrane were stained with Diff-Quick Staining kit (Biochemical Sciences, Swedesboro, NJ). The invaded cells were photographed randomly with microscope and quantified by counting the number of cells in three independent experiments.

### Small interfering RNA (siRNA) transfection

For transfection, the cells were grown to confluence in 6-cm plates and a Smad2 siRNA (Santa Cruz Biotechnology Inc.) and a ErbB3 siRNA at 60 pmol (Santa Cruz Biotechnology Inc.) were transfected using a siRNA transfection reagent (Santa Cruz Biotechnology Inc.) according to the manufacturer’s instructions. A nonspecific siRNA (Santa Cruz Biotechnology Inc.) was transfected as a control. After incubation for 6 h, the medium was replaced with the standard culture medium described above. After another 24 h of incubation, the transfected cells were treated with HRG-β1 and then used in subsequent evaluations.

### Statistical analysis

All experiments were performed in triplicate. The data were expressed as means ± SD. Statistical analyses were performed using Student’s *t*-test. Values of *P* < 0.05 were considered to indicate statistical significance.

## Results

### HRG-β1 induces Snail expression and EMT in SK-BR-3 and MCF7 cells

**Figure 1:**
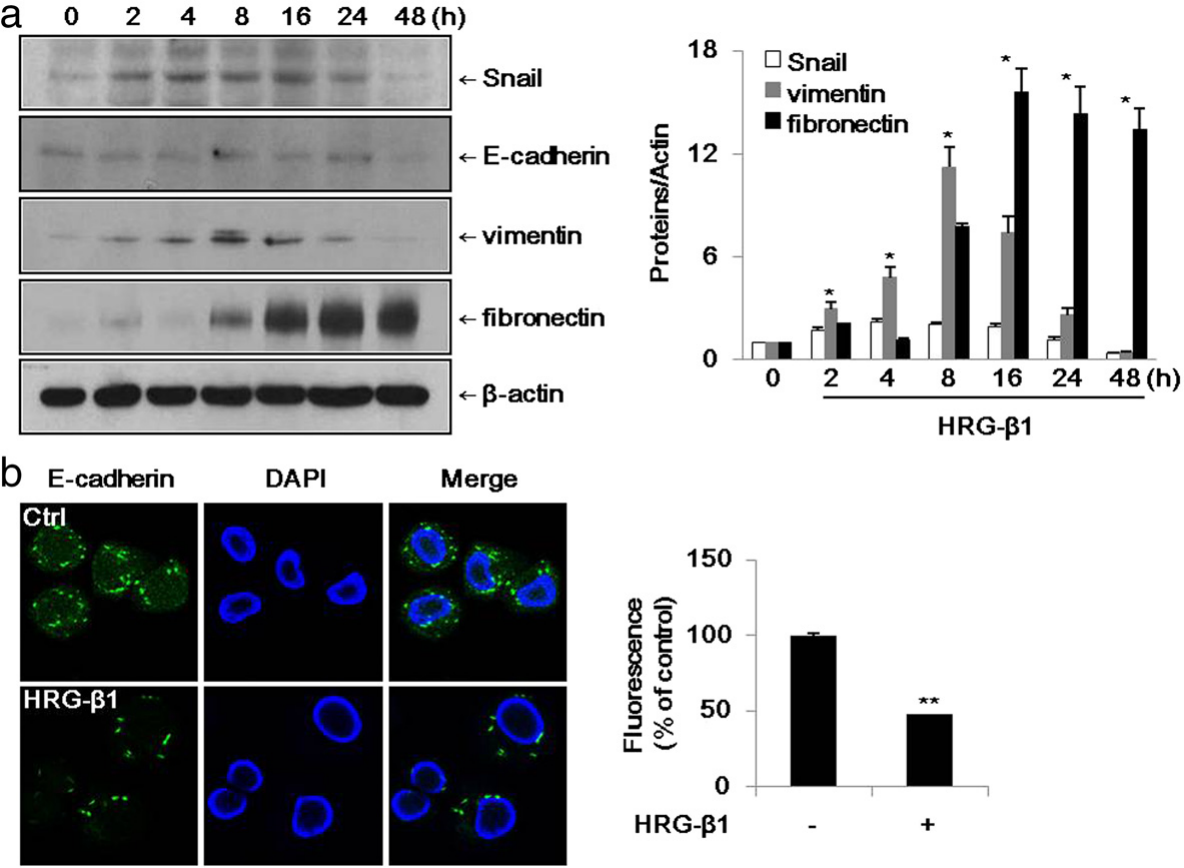
**HRG-β1 induces upregulation of the transcription factor Snail and EMT markers in SK-BR-3 cells. (a)** The cells were incubated with 25 ng/ml of HRG-β1 for different times after serum starvation. The expressions of Snail, mesenchymal markers including vimentin and fibronectin, and epithelial marker E-cadherin were examined by western blotting. β-actin was evaluated as a loading control. Data represent the means ± SD of three independent experiments. *P < 0.05, significant difference. **(b)** Immunofluorescence staining of E-cadherin protein. Cells were treated with or without 25 ng/ml of HRG-β1 for 48 h. The green color represents staining of E-cadherin and the blue color represents nuclear DNA staining by DAPI (magnification, ×400). The data were analyzed as the percentages of the control cells (**P < 0.01).

**Figure 2:**
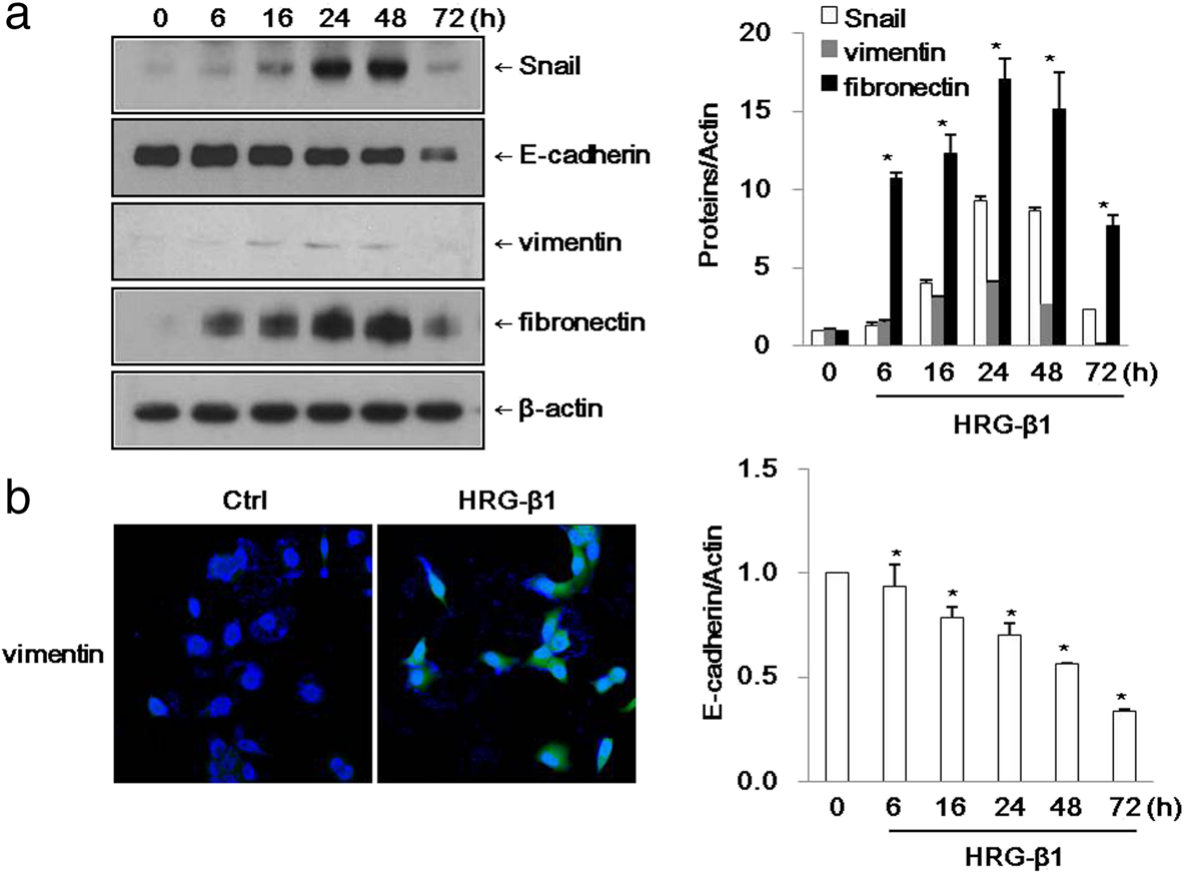
**HRG-β1 induces upregulation of the transcription factor Snail and EMT markers in MCF7 cells. (a)** The cells were incubated with 25 ng/ml of HRG-β1 for different times after serum starvation. The expressions of Snail, E-cadherin, vimentin, and fibronectin were examined by western blotting. β-actin was evaluated as a loading control. Data represent the means ± SD of three independent experiments. *P < 0.05, significant difference. **(b)** Immunofluorescence staining of vimentin protein. Cells were treated with or without 25 ng/ml of HRG-β1 for 24 h. The green color represents staining of vimentin and the blue color represents nuclear DNA staining by DAPI (magnification, ×200).

Cheng et al. have previously published that Snail is induced by HRG-β1 in SK-BR-3 cells [19]. As shown in Figure 1a, HRG-β1 increased the expression of Snail after 2 h and maintained its expression until 24 h in SK-BR-3 cells. We identified a few of the common acquired markers during EMT. Vimentin and fibronectin are commonly used to identify cells undergoing EMT in cancers. In SK-BR-3 cells, vimentin and fibronectin were expressed in a time-dependent manner after HRG-β1 treatment, while E-cadherin expression was decreased after 48 h of HRG-β1 treatment. We further examined the expression of E-cadherin by immunofluorescence staining, and found that E-cadherin was decreased in the HRG-β1-treated cells at 48 h compared with control cells (Figure 1b). In MCF7 cells, the expressions of Snail, vimentin, and fibronectin were increased after treatment with HRG-β1, while E-cadherin expression was suppressed at 72 h (Figure 2a). Immunofluorescence staining revealed that the expression of vimentin was increased in HRG-β1-treated cells compared with control cells (Figure 2b). These findings indicated that HRG-β1 upregulated Snail, vimentin, and fibronectin and suppressed E-cadherin in SK-BR-3 and MCF7 cells.

### HRG-β1 induces activation of Smad2 in SK-BR-3 and MCF7 cells

**Figure 3:**
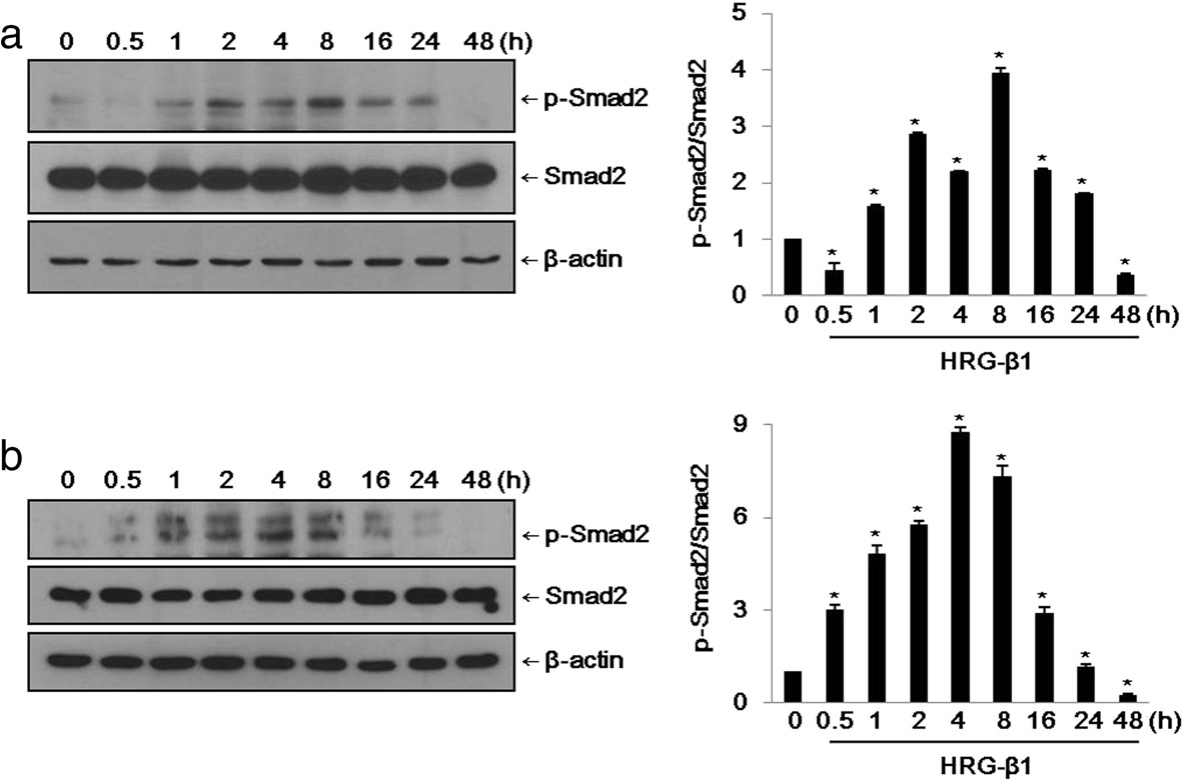
**HRG-β1 induces phosphorylation of Smad2 in SK-BR-3 (a) and MCF7 (b) cells. (a)** After 16 h of serum starvation in serum-free medium, SK-BR-3 cells were treated with 25 ng/ml of HRG-β1 for the indicated times. Immunoblots were probed with anti-phospho-Smad2 and anti-Smad2 antibodies. **(b)** The phosphorylation of Smad2 and total Smad2 were analyzed by western blotting in MCF7 cells. In all cases, β-actin was evaluated as a loading control. Data represent the means ± SD of three independent experiments. *P < 0.05, significant difference.

We examined the effects of the EGF family peptide HRG-β1 on the activation of Smad2 phosphorylation. HRG-β1 at 25 ng/ml induced the phosphorylation of Smad2 in a time-dependent manner in SK-BR-3 and MCF7 cells (Figure 3a, b). The level of phospho-Smad2 (Ser465/467) reached its maximum at 2–8 h after treatment and remained for 24 h without affecting the total Smad2 expression. Commonly, TGF-β1 induces phosphorylation of Smad2 within a few minutes of stimulation. Here, we found that HRG-β1 prolonged the phosphorylation of Smad2 compared with TGF-β1.

### Knockdown of ErbB3 expression suppresses HRG-β1-induced EMT in SK-BR-3 cells

**Figure 4:**
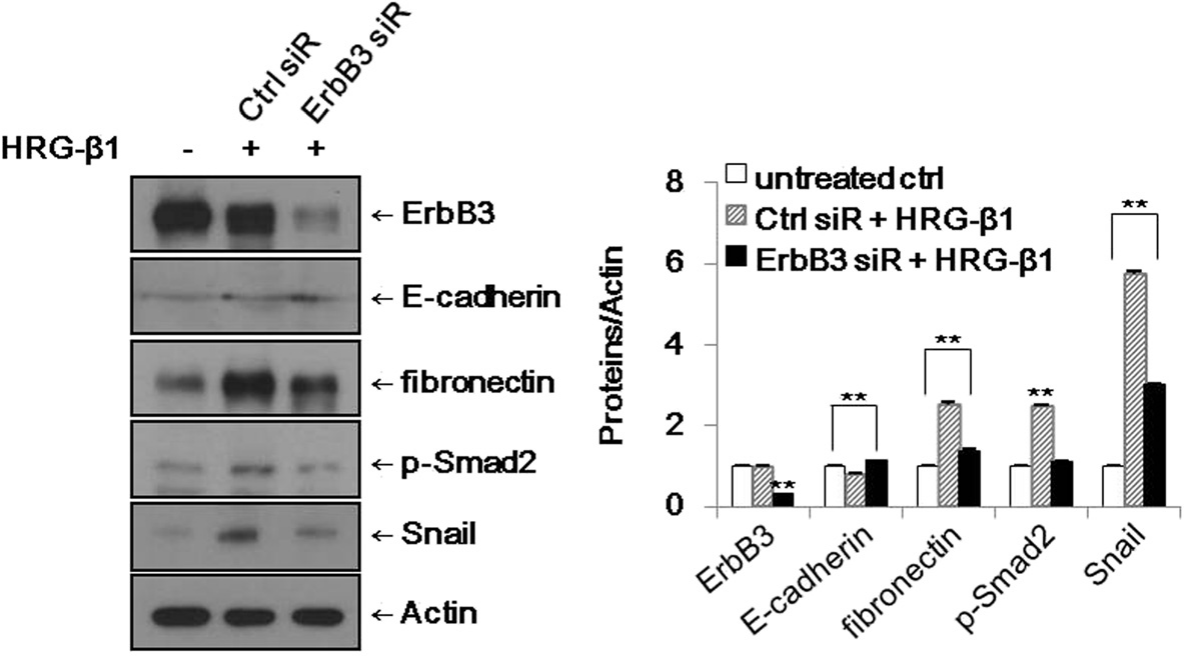
**Knockdown of ErbB3 suppresses HRG-β1-induced EMT in SK-BR-3 cells.** The cells were transfected with control (Ctrl) or ErbB3 siRNAs and treated with 25 ng/ml of HRG-β1 for 24 h. The expressions of ErbB3, E-cadherin, fibronectin, phospho-Smad2, and Snail were analyzed by western blotting. β**-**actin was reprobed as a loading control. Data represent the means ± SD of three independent experiments. *P < 0.05, **P < 0.01, significant difference.

As shown in Figure 4, knockdown of ErbB3 expression by siRNA transfection suppressed the expressions of phospho-Smad2, Snail, and fibronectin by HRG-β1, whereas the expression of E-cadherin was increased in ErbB3 siRNA-transfected cells (ErbB3 siR) compared with control siRNA-transfected SK-BR-3 cells (Ctrl siR). On this basis, HRG-β1/ErbB3 signaling induced EMT in the SK-BR-3 and MCF7 breast cancer cell lines.

### HRG-β1 induces expression of Snail through activation of Smad2 via the PI3k/Akt signaling pathway

**Figure 5:**
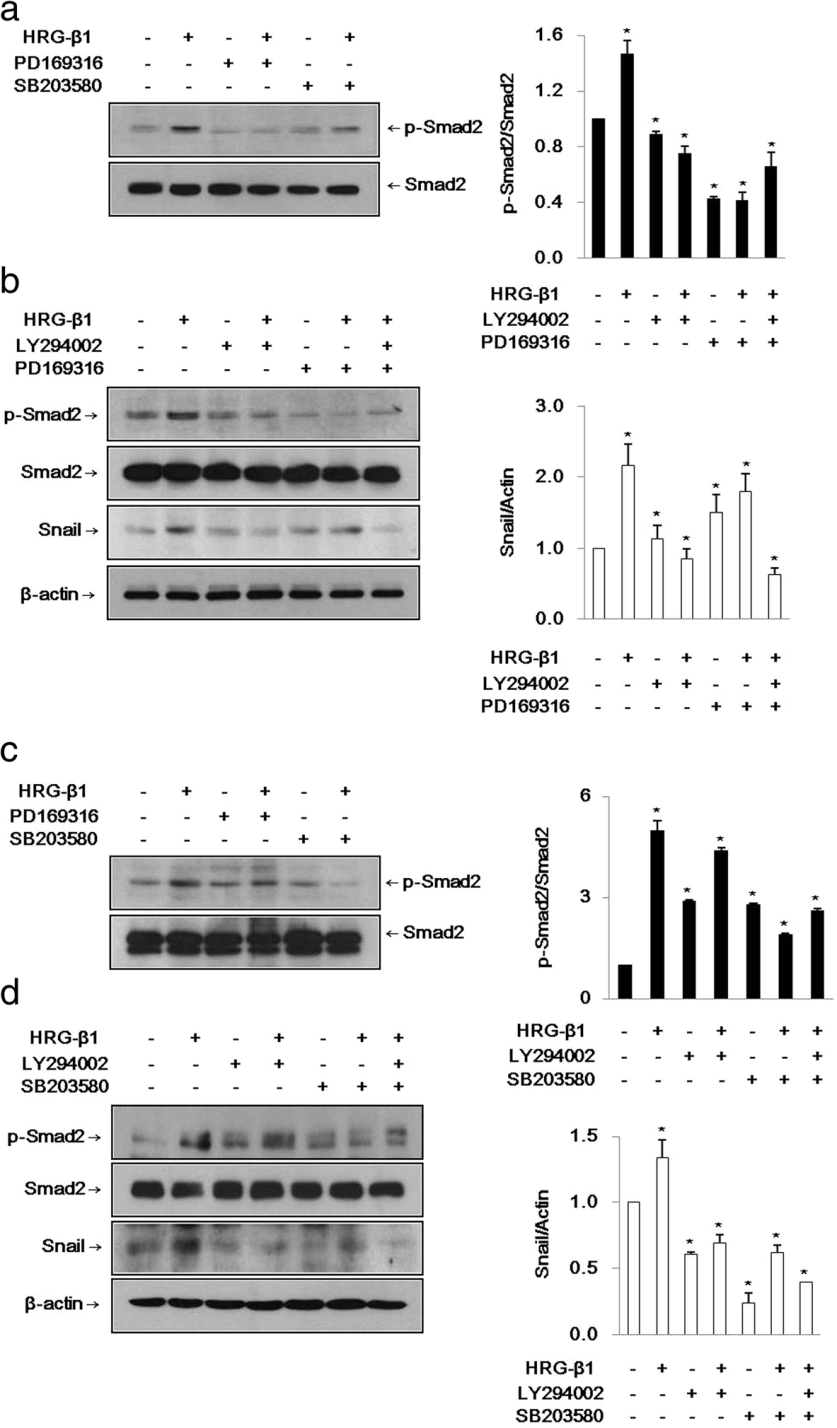
**HRG-β1 induces expression of Snail through phospho-Smad2 via PI3k/Akt in SK-BR-3 (a, b) and MCF7 (c, d) cells. (a, c)** The cells were pretreated with vehicle (DMSO) as a control or 10 μM of phospho-Smad2 pharmacological inhibitors, PD169316 or SB203580 for 1 h, and then stimulated with HRG-β1 for 24 h. The inhibition of phospho-Smad2 by the inhibitors and total Smad2 were analyzed by western blotting in SK-BR-3 and MCF7 cells. **(b)** SK-BR-3 cells were pretreated with vehicle or 10 μM of LY294002 or PD169316 prior to HRG-β1 stimulation for 24 h. The cells were harvested and immunoblots were analyzed with anti-phospho-Smad2, anti-Smad2, anti-Snail, and anti-β-actin antibodies. **(d)** Inhibition of HRG-β1-induced phospho-Smad2 and Snail expressions by 10 μM of LY294002 or SB203580 without affecting total Smad2 and constitutive β-actin expressions in MCF7 cells. Data represent the means ± SD of three independent experiments. *P < 0.05, significant difference.

First, we identified that HRG-β1-induced Smad2 phosphorylation was inhibited by pretreatment with the PI3k inhibitor LY294002 (data not shown). It is known that HRG-β1 phosphorylates Smad2 via the PI3k/Akt signaling pathway [19]. Therefore, to investigate the possible involvement of Smad2 in HRG-β1-induced Snail gene expression, SK-BR-3 and MCF7 cells were pretreated with two known inhibitors of Smad2 phosphorylation, PD169316 and SB203580 [20,21]. PD169316 inhibited HRG-β1-induced Smad2 phosphorylation in SK-BR-3 cells and SB203580 had a more efficient inhibitory effect in MCF7 cells (Figure 5a, c). We pretreated the cells with LY294002, PD169316, or SB203580 alone and combinations of LY294002 and PD169316 or SB203580 prior to HRG-β1 stimulation to both cell types. As shown in Figure 5b, d, the HRG-β1-induced expressions of phospho-Smad2 and Snail were inhibited by treatment with the above inhibitors, indicating that HRG-β1 induced expression of Snail through activation of Smad2 via the PI3k/Akt signaling pathway. Since these Smad2 phosphorylation inhibitors are also known to block p38 phosphorylation, the role of Smad2 was further explored by the more specific genetic approach of RNA interference (siRNA).

### HRG-β1 induces nuclear colocalization of phospho-Smad2 and Snail

**Figure 6:**
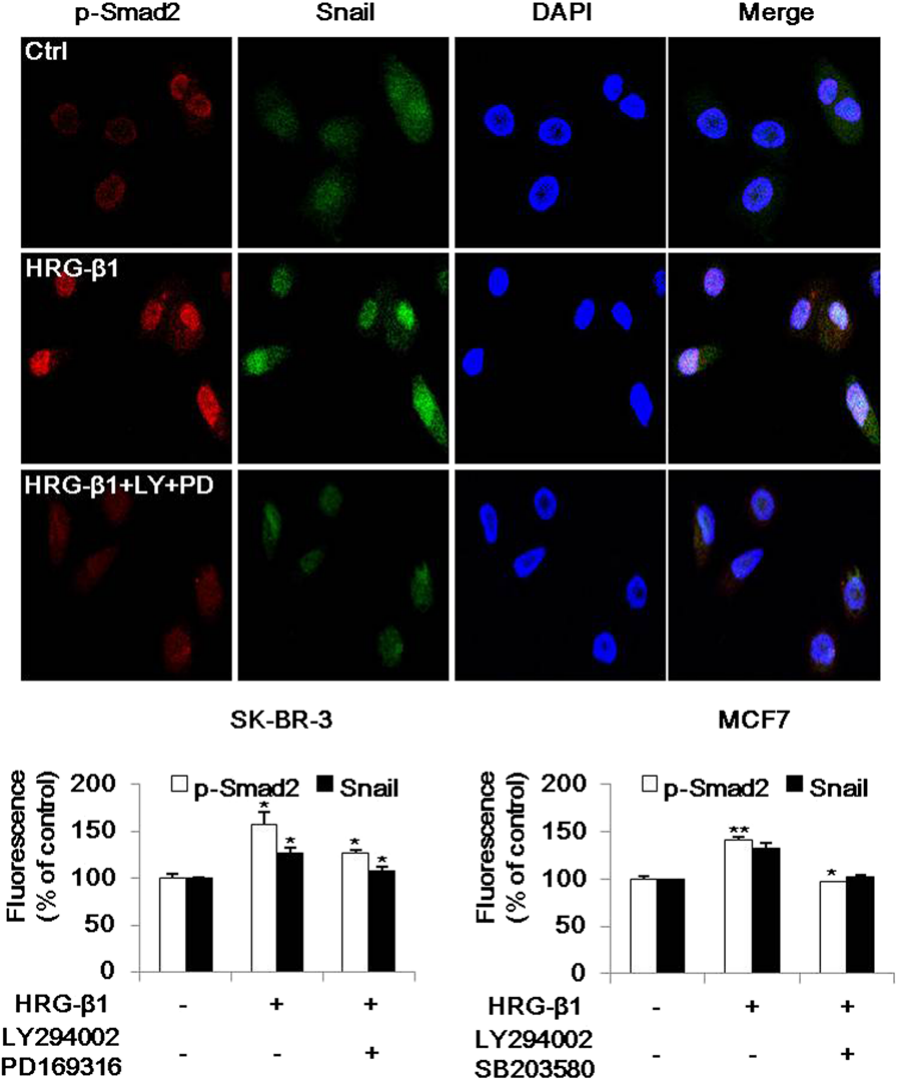
**HRG-β1 induces nuclear colocalization of phospho-Smad2 and Snail.** Immunofluorescence analyses were performed for the nuclear colocalization of phospho-Smad2 and Snail in SK-BR-3 cells. Cells were pretreated with vehicle or 10 μM of LY294002 or PD169316 for 1 h prior to stimulation with 25 ng/ml of HRG-β1. After incubation for a further 24 h, phospho-Smad2 (red) and Snail (green) were observed under a confocal laser scanning microscope and the nuclear DNA was stained with DAPI (blue; magnification, ×200). The lower graphs show the fluorescence intensities of phospho-Smad2 and Snail as percentages compared with control cells in three independent experiments involving SK-BR-3 (left) and MCF7 (right) cells. *P < 0.05, **P < 0.01, significant difference.

HRG-β1 treatment for 24 h induced nuclear colocalization of phospho-Smad2 and Snail in SK-BR-3 cells, and this translocation to the nucleus was inhibited by pretreatment with LY294002 and PD169316 before HRG-β1 stimulation (Figure 6). In MCF7 cells, HRG-β1 induced nuclear colocalization of phospho-Smad2 and Snail, and pretreatment with LY294002 and SB203580 suppressed the nuclear translocation induced by HRG-β1 (data not shown). The mean percentages of fluorescence of phospho-Smad2 and Snail are also shown in Figure 6.

### HRG-β1 induces EMT through phospho-Smad2-mediated Snail via the PI3k/Akt signaling pathway

**Figure 7:**
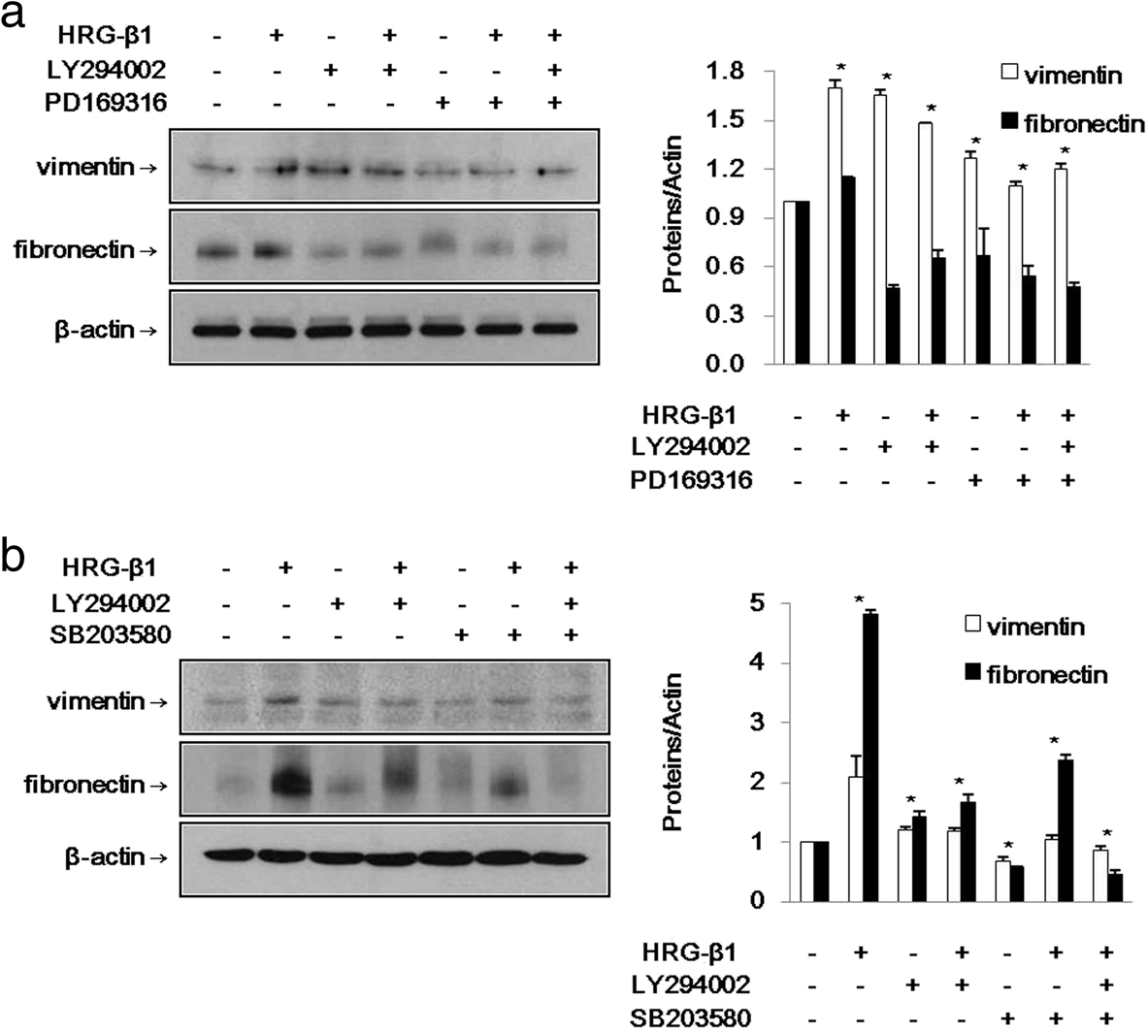
**HRG-β1 induces EMT through phospho-Smad2-mediated Snail via PI3k/Akt signaling in SK-BR-3 (a) and MCF7 (b) cells. (a)** The cells were pretreated for 1 h with vehicle, 10 μM of LY294002, or PD169316 alone or a combination of LY294002 and PD169316 and then treated with 25 ng/ml of HRG-β1. The expressions of vimentin and fibronectin were identified by western blotting. (**b**) The cells were treated with 25 ng/ml of HRG-β1 after pretreatment with 10 μM of LY294002 or SB203580 alone or a combination of LY294002 and SB203580. Immunoblots were probed with anti-vimentin and anti-fibronectin antibodies. In all cases, β-actin served as a loading control. Data represent the means ± SD of three independent experiments. *P < 0.05, significant difference.

As mentioned earlier, HRG-β1 increased the expressions of vimentin and fibronectin during EMT in SK-BR-3 and MCF7 cells. As shown in Figure 7a, b, the HRG-β1-induced expressions of vimentin and fibronectin were inhibited by the indicated inhibitors. Taken together, HRG-β1 induced EMT through phospho-Smad2-mediated expression of Snail via the PI3k/Akt signaling pathway in both breast cancer cell lines.

### Knockdown of Smad2 expression suppresses HRG-β1-induced expressions of Snail and fibronectin

**Figure 8:**
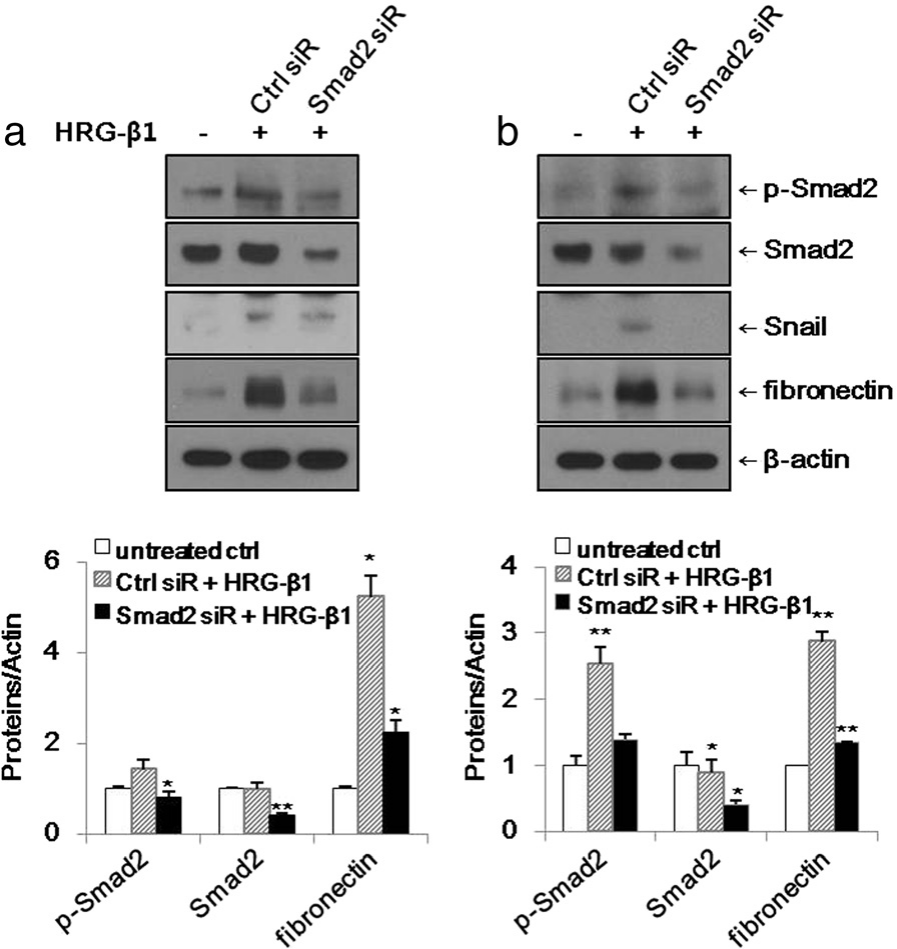
**Knockdown of Smad2 suppresses HRG-β1-induced expressions of Snail and fibronectin in SK-BR-3 (a) and MCF7 (b) cells. (a, b)** The cells were transfected with control or Smad2 siRNAs prior to treatment with 25 ng/ml of HRG-β1. After incubation for a further 24 h, the expressions of phospho-Smad2, Smad2, Snail, and fibronectin were analyzed by western blotting. β**-**actin was reprobed as a loading control. Data represent the means ± SD of three independent experiments. *P < 0.05, **P < 0.01, significant difference.

SK-BR-3 and MCF7 cells were transfected with control and Smad2 siRNAs. As shown in Figure 8a, b, the HRG-β1-increased expressions of Snail and fibronectin in control siRNA-transfected cells (Ctrl siR) compared with untreated control cells (untreated ctrl) were downregulated in Smad2 siRNA-transfected cells (Smad2 siR). Taken together, Smad2 activation plays roles in the expression of Snail and induction of EMT by HRG-β1 in SK-BR-3 and MCF7 cells.

### HRG-β1 and ErbB3 induces cancer cell migration and invasion through Smad2 activation

**Figure 9:**
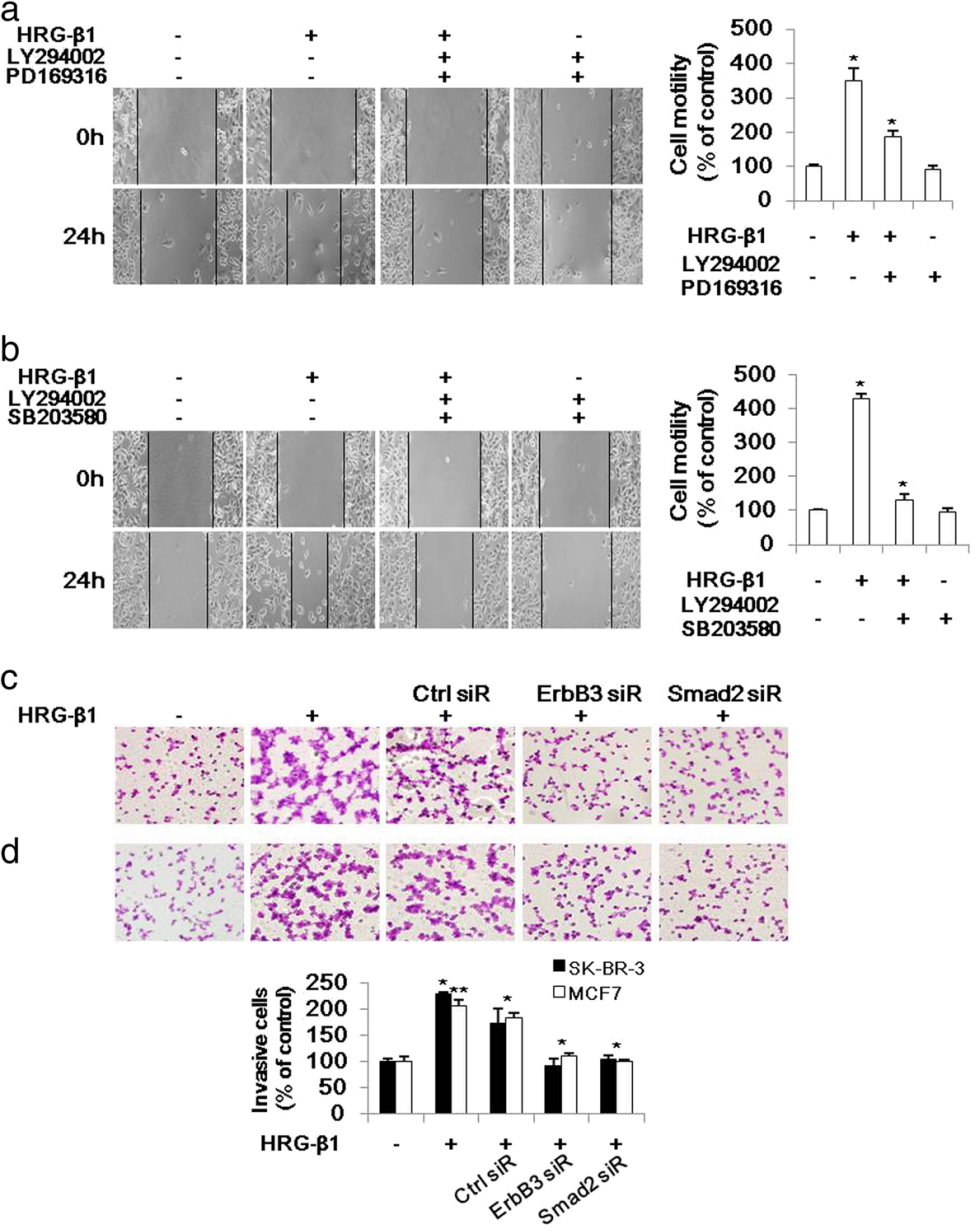
**HRG-β1-induces cancer cell migration and invasion through Smad2 activation in SK-BR-3 (a, c) and MCF7 (b, d) cells. (a, b)** The motility of each cell type was assessed by wound healing assays. A scratch was made across confluent monolayers using a plastic tip and the cells were then pretreated with 10 μM of LY294002 and PD169316 or SB203580 prior to stimulation with HRG-β1. After 24 h of incubation, the migrated cells were monitored using a light microscope. Data were analyzed as percentages of the control cells in three independent experiments. *P < 0.05, significant difference. **(c, d)** A matrigel invasion assay was used to quantify cell invasion. After 24 h of transfection, the cells were seeded into upper chambers and incubated for 48 h in the presence of 25 ng/ml of HRG-β1. Then, the cells that invaded into the lower surface were photographed under a light microscope, with × 200 magnification. Data were analyzed as the percentage of the control of the three independent experiments. *P < 0.05 and **P < 0.01 were considered significant.

We performed in vitro wound healing assays. Pretreatment with LY294002 and PD169316 or SB203580 inhibited the cell migration of SK-BR-3 and MCF7 cells in the presence of HRG-β1 (Figure 9a, b). In cell invasion assay, knockdown of ErbB3 and Smad2 by siRNA transfection inhibited the cell invasive ability of SK-BR-3 and MCF7 cells under HRG-β1 stimulation in matrigel-coated chamber (Figure 9c, d). Collectively, these data suggested that HRG-β1 induced cancer cell migration and invasion through induction of EMT via PI3k/Akt-phospho-Smad2-Snail signaling pathway.

## Discussion

Breast cancer is the most common malignancy among women worldwide. Understanding the mechanisms of cancer invasion and metastasis is a very important issue in cancer research. The majority of studies regarding EMT have focused on TGF-β signaling in various kinds of disease settings [5,6,8,22]. Thus far, the basal-like type and triple-negative type of breast carcinomas are characterized to show mesenchymal and stem-cell features and are known to be correlated with resistance to therapy [23,24].

It has been suggested that not only TGF-β but also various kind of signaling molecules, such as growth factors, cytokines, integrins, and Wnts, are inducers of EMT [25]. HRG is a ligand for ErbB3 and ErbB4 and has also been reported to promote the invasive behavior of breast cancer cells in vitro [26]. HRG-induced ErbB2/ErbB3 heterodimers are considered to induce strong downstream signaling and to activate various biological responses, such as cellular proliferation, maturation, survival, apoptosis, and angiogenesis [27-31]. Cheng et al. [19] demonstrated that HRG-β1 induced EMT through Snail upregulation via the PI3k/Akt pathway in the ErbB2-overexpressing SK-BR-3 cell line. Various kinds of cancer cells, such as breast cancer cells, glial cells, neural tissues, and hepatocytes, are known to secrete HRG [32]. Although the tumor cells can be stimulated by HRG in autocrine or paracrine manners, small numbers of circulating tumor cells can be activated by nearby HRG-secreting organs, such as the liver and central nervous system, where cancer cells move to and settle down. Blockade of HRG expression inhibits tumorigenesis and metastasis of breast cancer cells [33].

In this study, we have obtained evidence that HRG plays an important role in breast cancer.

It is a novel observation that the induction of EMT by HRG-β1 via upregulation of Snail involved the Smad2 signaling pathway, which is one of TGF-β signaling molecules. We found that phospho-Smad2 inhibitors (PD169316 or SB203580) and Smad2 siRNA transfection inhibited Snail expression and EMT, which were induced by HRG-β1. Furthermore, we identified that HRG-β1 induced cancer cell migration and invasion through Smad2 activation by wound healing assays and matrigel invasion assays. Overall, HRG-β1 induced EMT through Snail expression by activation of Smad2 not only in the SK-BR-3 cell line, but also in the MCF7 cell line, which expresses ErbB2 at basal levels. This dynamic and reversible emergence of the mesenchymal phenotype can be triggered by a variety of tumor microenvironments in the non-basal-like phenotypes of breast cancer cell lines.

Activation of RTK signaling caused by HRG-associated heterodimerization of ErbB3 and ErbB2 may be a critical step in tumor progression. We identified that the ErbB2 interaction with ErbB3 is required for the HRG-β1-induced EMT process. Specific siRNA transfection is a useful tool for evaluating the biologic effects of a target gene. In the presence of HRG-β1, knockdown of ErbB3 resulted in suppression of phospho-Smad2, Snail, and fibronectin expressions, whereas the expression of E-cadherin was increased in SK-BR-3 cells. Taken together, ErbB3 contributed to the HRG-β1-induced EMT process and cell migration through phospho-Smad2-mediated expression of Snail via the PI3k/Akt signaling pathway in SK-BR-3 and MCF7 breast cancer cells.

These findings are important for defining the tumorigenic roles of ErbB receptors and HRG as well as Smad2 activation in breast cancers, because HRG-β1 can overcome the inhibitory effects of anti-EGFR therapies on cell growth and activate invasion in tamoxifen-resistant cells through promotion of ErbB3/ErbB2 heterodimerization and activation of the PI3k/Akt signaling pathway [34].

## Conclusions

In conclusion, we have demonstrated a downstream signal transduction pathway of HRG-β1-induced EMT that occurred in the SK-BR-3 and MCF7 breast cancer cell lines. Therefore, we suggest that blockade of the EMT mechanisms by HRG, including ErbB3 and not only Snail but also Smad2, might be a useful therapeutic target in breast cancer.

## Competing interests

The authors declare that they have no competing interests.

## Authors’ contributions

JK performed the laboratory work and the statistical analysis. AK participated in the design of the study. HJ, YL, CK, and HK participated in its design and coordination and helped to draft the manuscript. JK and AK wrote the manuscript. CK critically revised the manuscript. All authors read and approved the final version of the manuscript.

## Pre-publication history

The pre-publication history for this paper can be accessed here:

http://www.biomedcentral.com/1471-2407/13/383/prepub
